# Molecular Mechanisms Influencing Bacterial Conjugation in the Intestinal Microbiota

**DOI:** 10.3389/fmicb.2021.673260

**Published:** 2021-06-04

**Authors:** Kevin Neil, Nancy Allard, Sébastien Rodrigue

**Affiliations:** Départment de Biologie, Université de Sherbrooke, Sherbrooke, QC, Canada

**Keywords:** bacterial conjugation, microbiota, conjugative plasmids (CP), mating pair stabilization, antibiotic resistance

## Abstract

Bacterial conjugation is a widespread and particularly efficient strategy to horizontally disseminate genes in microbial populations. With a rich and dense population of microorganisms, the intestinal microbiota is often considered a fertile environment for conjugative transfer and a major reservoir of antibiotic resistance genes. In this mini-review, we summarize recent findings suggesting that few conjugative plasmid families present in *Enterobacteriaceae* transfer at high rates in the gut microbiota. We discuss the importance of mating pair stabilization as well as additional factors influencing DNA transfer efficiency and conjugative host range in this environment. Finally, we examine the potential repurposing of bacterial conjugation for microbiome editing.

## Introduction

Antimicrobial resistance continues to rise worldwide, with alarming projections suggesting that antibiotic-resistant infections could become the second most common cause of death by 2050 (O’ Neil, 2014). This led many research groups to study the global collection of antibiotic resistance genes, also called the resistome (Carattoli, 2013; Penders et al., 2013; van Schaik, 2015; Casals-Pascual et al., 2018), and to identify the intestinal microbiota as a major reservoir of antibiotic resistance genes (Ravi et al., 2014). The complex microbial communities found in the gut are dense and composed of diverse bacteria phyla (Turnbaugh et al., 2007; Qin et al., 2010), a context thought to be particularly favorable for horizontal gene transfer (Liu et al., 2012; Soucy et al., 2015) and antibiotic resistance gene dissemination (San Millan, 2018). Given that the intestinal microbiota also contains a variety of pathobionts (Palleja et al., 2018; Bakkeren et al., 2019), understanding the molecular mechanisms driving the spread of antibiotic resistance genes is particularly important to prevent infections that could become difficult or impossible to treat.

Horizontal gene transfer mechanisms include transformation, transduction, and bacterial conjugation. Bacterial conjugation is considered a major contributor to gene transfer and to the emergence of new antibiotic-resistant pathogens. Conjugative transfer is a well characterized phenomenon during which a donor bacterium assembles a type IV secretion system (T4SS) and transfers DNA to a recipient bacterium in close contact (Cascales and Christie, 2003; Alvarez-Martinez and Christie, 2009; Arutyunov and Frost, 2013; Virolle et al., 2020). Although thoroughly investigated in test tubes and Petri dishes, the study of bacterial conjugation in the intestinal microbiota remains far less characterized with most evidence being provided by epidemiologic studies (Norman et al., 2009; Chen et al., 2013; Soucy et al., 2015; Sun et al., 2016; San Millan, 2018). IncF, IncI, IncA, IncC, and IncH plasmids are the most frequently encountered in humans and animals (Rozwandowicz et al., 2018) but few studies have quantified the transmission of mobile genetic elements *in situ* and described the underlying mechanisms. This mini-review summarizes recent findings on bacterial conjugation in the gut microbiome with a focus on enterobacteria.

## The Mobility of Genes in the Gut Microbiota

Many studies have reported conjugative transfer of plasmids in the intestinal microbiota (Licht and Wilcks, 2005). For instance, conjugation was found to occur with plasmids of different incompatibility groups (Table 1) harbored by Gram-negative (Kasuya, 1964; Reed et al., 1969; Jones and Curtiss, 1970; Duval-Iflah et al., 1980, 1994; Corpet et al., 1989; Garrigues-Jeanjean et al., 1999; Licht et al., 1999, 2003; García-Quintanilla et al., 2008; Stecher et al., 2012; Aviv et al., 2016; Gumpert et al., 2017; Bakkeren et al., 2019; Neil et al., 2020; Ott et al., 2020) or Gram-positive bacteria (Doucet-Populaire et al., 1991, 1992; McConnell et al., 1991; Schlundt et al., 1994; Igimi et al., 1996; Jacobsen et al., 1999; Moubareck et al., 2003; Lester et al., 2004). Most studies focused on *Escherichia coli* as the donor bacterium but lactic acid bacteria have also been investigated because of their abundance in fermented food products (Igimi et al., 1996). Despite major implications on microbial evolution and on the emergence of antibiotic-resistant pathogens, our knowledge of the molecular mechanisms facilitating bacterial conjugation in the gut microbiota remains sparse (Norman et al., 2009; Soucy et al., 2015; Casals-Pascual et al., 2018).

**TABLE 1:** Transfer rates of various conjugative elements in the intestinal microbiota.

**Name**	**Inc group**	**Resistance**	**Isolated in**	**Donor strain**	**Recipient strain**	Transfer rates†in vitro**	**Transfer rates†*in situ***	**MPS family**	**Genbank**	**References**
pAMβ1	18	Er, Lc	*Enterococcus faecalis*	*Lactococcus lactis* IL1403	*Enterococcus faecalis* HS32	2.3 × 10^–3^	<1 × 10^–7^ (a)	Not reported	NC_ 013514.1	Igimi et al., 1996
pAT191 (synthetic)*	18	Km	*Enterococcus faecalis*	*Enterococcus faecalis* BM4110	*Escherichia coli* K802N::Tn10	5 × 10^–9^	3 × 10^–9^ (a)	Not reported	Not deposited	Doucet-Populaire et al., 1992
pAM714	HIy	Er	*Enterococcus faecalis*	*Enterococcus faecalis* FA2-2	*Enterococcus faecalis* JH2SS	∼1 × 10^–2^	1.4 × 10^–1^ (b)	Not reported	Not deposited	Huycke et al., 1992
pAM771	HIy	Er	*Enterococcus faecalis*	*Enterococcus faecalis* FA2-2	*Enterococcus faecalis* JH2SS	Not reported	2.9 × 10^–2^ (b)	Not reported	Not deposited	Huycke et al., 1992
pCAL1/pCAL2	Not found	Er	*Enterococcus faecium*	*Enterococcus faecium* 160/00	*Enterococcus faecium* 64/3-RFS	2 × 10^–5^	∼1 × 10^–6^ (a)	Not reported	Not deposited	Lester et al., 2004
pCF10	Not found	Tc	*Enterococcus faecalis*	*Enterococcus faecalis* OG1RFS	*Enterococcus faecalis* OG1SS	Data not shown	∼1 × 10^–3^ (c)	Not reported	NC_ 006827.2	Licht et al., 2002
*Tn1545*	–****	Km, Er, Tc	*Streptococcus pneumoniae*	*Enterococcus faecalis* BM4110	*Listeria monocytogenes* LO17RF	2.5 × 10^–7^	1.1 × 10^–8^ (a)	Not reported	AM903082.1	Doucet-Populaire et al., 1991
*Tn916*	–****	Tc	*Bacillus subtilis*	*Enterococcus faecalis* OG1SS	*Enterococcus faecalis* OG1RF	1.1 × 10^–5^	∼1 × 10^–9^ (d)	Not reported	KM516885.1	Bahl et al., 2004
pYD1	Not found	14 antibiotic resistance markers	*Serratia liquefasciens*	*Serratia liquefasciens*	*Escherichia coli*	Not reported	∼1 × 10^–6^ (a)	Not reported	Not deposited	Duval-Iflah et al., 1980
ROR-1	Not found	Tc	Not found	*Escherichia coli* M7-18	*Escherichia coli* x820	∼1 × 10^–5^	∼1 × 10^–4^ (a)	Not reported	Not deposited	Jones and Curtiss, 1970
pIP72	B/O	Km	*Escherichia coli*	*Escherichia coli* Nissle1917	*Escherichia coli* Nissle1917	3.57 × 10^–4^	3.56 × 10^–5^ (a)	PilV	MN612051.1	Neil et al., 2020
pVCR94ΔX3	C	Km	*Vibrio cholerae*	*Escherichia coli* Nissle1917	*Escherichia coli* Nissle1917	3.23 × 10^–3^	Not detected (a)	TraN	KF551948.1	Neil et al., 2020
pSLTΔfinO	F	Km	*Salmonella enterica* subsp. *enterica* serovar Typhimurium	*Salmonella enterica* subsp. *enterica* serovar Typhimurium SV5535	*Salmonella enterica* subsp. *enterica* serovar Typhimurium SV5534	5 × 10^–4^	5 × 10^–5^ (a)	TraN	AE006471.2	García-Quintanilla et al., 2008
pOX38	FI	Sp, Tc, Su	*Escherichia coli*	*Escherichia coli* Nissle1917	*Escherichia coli* Nissle1917	6.82 × 10^–2^	4.89 × 10^–5^ (a)	TraN	MF370216.1	Neil et al., 2020
RIP71a	FII	Ap, Tc, Cm, Sm, Sp	*Escherichia coli*	*Escherichia coli* Nissle1917	*Escherichia coli* Nissle1917	2.64 × 10^–3^	7.87 × 10^–4^ (a)	TraN	MN626601	Neil et al., 2020
R1	FII	Km, Cm, Su, Sp	*Salmonella enterica* subsp. *enterica* serovar Paratyphi B	*Escherichia coli* Nissle1917	*Escherichia coli* Nissle1917	2.97 × 10^–3^	1.6 × 10^–4^ (a)	TraN	KY749247.1	Neil et al., 2020
R1drd19	FII	Km, Cm, Su, Sp, Ap	*Salmonella enterica* subsp. *enterica* serovar Paratyphi B	*Escherichia coli* BJ4	*Escherichia coli* BJ4	∼1 × 10^–1^	∼1 × 10^–3^ (a)	TraN	Not deposited	Licht et al., 1999
pCVM29188_146	FIIA	Sm, Tc	*Salmonella enterica* subsp. *enterica* serovar Kentucky	*Salmonella enterica* subsp. *enterica* serovar Kentucky	*Escherichia coli* HS-4	∼1 × 10^–4^	∼5 × 10^–4^ (a)	TraN	CP001122.1	Ott et al., 2020
TP123	HI1	Sm, Cm, Su, Sp	*Salmonella enterica* subsp. *enterica* serovar Typhi	*Escherichia coli* Nissle1917	*Escherichia coli* Nissle1917	8.05 × 10^–3^	Not detected (a)	TraN	MN626602.1	Neil et al., 2020
R64	I1α	Sm, Tc	*Salmonella enterica* subsp. *enterica* serovar Typhimurium	*Escherichia coli* Nissle1917	*Escherichia coli* Nissle1917	5.51 × 10^–4^	1.54 × 10^–6^ (a)	PilV	NC_ 005014.1	Neil et al., 2020
p2kan	I1	Km	*Salmonella enterica* subsp. *enterica* serovar Typhimurium	*Salmonella enterica* subsp. *enterica* serovar Typhimurium SL1344	*Escherichia coli*	7.53 × 10^–3^ to 5.20 × 10^–9^	∼1 × 10^0^ (a)	PilV	Not deposited	Stecher et al., 2012
pHUSEC41-1	I1	Su, Ap, Sm, Pip	*Escherichia coli* HUSEC41	*Escherichia coli*	*Escherichia coli*	Not reported	Not reported (a)	PilV	NC_ 018995.1	Gumpert et al., 2017
pES1	I1	Tc, Su, Tr	*Salmonella enterica* subsp. *enterica* serovar Infantis	*Escherichia coli*	*Salmonella enterica* subsp. *enterica* serovar Typhimurium SL1344	1.2 × 10^–6^	2 × 10^–7^ (a)	PilV	NZ_ CP047882.1	Aviv et al., 2016
TP114	I2	Km	*Escherichia coli*	*Escherichia coli* Nissle1917	*Escherichia coli* Nissle1917	7.05 × 10^–3^	1.12 × 10^–1^ (a)	PilV	MF521836.1	Neil et al., 2020
pIP69	L/M	Ap, Km, Tc	*Salmonella enterica* subsp. *enterica* serovar Paratyphi	*Escherichia coli* Nissle1917	*Escherichia coli* Nissle1917	9.73 × 10^–7^	Not detected (a)	Not reported	MN626603	Neil et al., 2020
RP1/RP4	P1α	Ap, Km, Tc	*Pseudomonas aeruginosa*	*Escherichia coli* HB101	*Escherichia coli* X7	2.05 × 10^–1^	9.21 × 10^–5^ (a)	None	BN000925.1	Rang et al., 1996
				*Escherichia coli* BJ4	*Escherichia coli* BJ4	2.56 × 10^–2^	Not detected*** (a)	None	BN000925.1	Licht et al., 2003
pRK24 (derived from RK2)		Ap, Tc	*Enterobacter aerogenes*	*Escherichia coli* Nissle1917	*Escherichia coli* Nissle1917	4.07 × 10^–1^	Not detected (a)	None	Not deposited	Neil et al., 2020
pRts1	T	Km, Sp	*Proteus vulgaris*	*Escherichia coli* Nissle1917	*Escherichia coli* Nissle1917	2.63 × 10^–4^	Not detected (a)	TraN	MN626604	Neil et al., 2020
R388	W	Su, Tm	*Escherichia coli*	*Escherichia coli* Nissle1917	*Escherichia coli* Nissle1917	3.09 × 10^–4^	Not detected (a)	None	NC_028464	Neil et al., 2020
				*Escherichia coli* UB1832	*Escherichia coli* UB281	∼1 (10^0^)	∼1 × 10^–4^ (a)	None	NC_028464	Duval-Iflah et al., 1994, 1998
R6K	X2	Ap, Sm	*Escherichia coli*	*Escherichia coli* Nissle1917	*Escherichia coli* Nissle1917	1.21 × 10^–2^	2.5 × 10^–4^ (a)	Not reported	LT827129.1	Neil et al., 2020

*Derived from pAMβ1 conjugative plasmid, has pBR322 origin of replication.

**Measure taken for conjugation on agar plate.

***In conditions not selecting for transconjugants.

****Conjugative transposons integrate into the chromosome of their host, and hence, plasmid incompatibility groups do not apply.

*^†^Transconjugants/recipients.*

*(a) Mice model; (b) Hamster model; (c) Pig model; (d) rat model.*

*MPS family is indicated as “not reported” when the exact mechanism has not been described or with “none” when experimental evidence show that this function is absent.*

Several environmental conditions resembling those encountered in the intestinal tract were investigated *in vitro* and shown to influence conjugation (Rang et al., 1996). For example, the transfer rates of conjugative plasmids pES1 and pSLT were shown to be affected by lower oxygen levels and the presence of bile salt or by other factors such as NaCl concentration and temperature (García-Quintanilla et al., 2008; Aviv et al., 2016). Other plasmids were shown to be inhibited by the presence of mammalian cells in co-cultures, raising the possibility that human host secreted factors could affect plasmid transfer rates (Lim et al., 2008; Machado and Sommer, 2014). A pioneering study reported in 1999 that IncF plasmid R1drd19 can transfer between two *E. coli* strains within the mouse gut microbiome at rates similar to those obtained on agar plates (Licht et al., 1999). This led to the hypothesis that bacterial mating may occur in a stable matrix, most likely after the formation of biofilm in the gut. *In situ* transfer rates were also quantified directly in the mouse intestinal microbiota for other conjugative plasmids (Table 1). However, different models with several experimental variables were used. For example, different mice models ranging from germ-free to antibiotic-treated mice have been reported (Licht and Wilcks, 2005). Another important variable comes from the nature of the donor strain and recipient strains, which were shown to affect transfer rates in the gut (Ott et al., 2020). While some studies introduced and probed specific bacteria as recipient cells for conjugation, other investigations used endogenous residents of the microbiota. Furthermore, mixing donors and recipient strains before their introduction in the mice (Stecher et al., 2012) could also introduce differences since conjugation could occur between the two strains before or in the stomach immediately after their introduction in mice rather than in the intestinal microbiota. Taken together, these variations in experimental models make the comparison of transfer rates difficult between studies.

A recent study by our group adopted a standardized assay to evaluate and compare the mobility of conjugative plasmids in the mouse gut microbiota (Neil et al., 2020). Transfer rates were quantified for 13 conjugative plasmids representing 10 of the major conjugative plasmids incompatibility groups found in *Enterobacteriaceae* (Table 1). This work was performed in streptomycin-treated mice to deplete endogenous enterobacteria and facilitate the establishment of *E. coli* Nissle, 1917 derivatives as the donor and recipient bacteria. This work revealed that few conjugative plasmids were able to efficiently transfer *in situ* using this model, without any correlation with *in vitro* conjugation rates. A surprising finding was that incompatibility group I_2_ (IncI_2_) plasmid TP114 displayed only modest conjugation efficiencies *in vitro* but reached very high transfer rates in the intestinal microbiota, which prompted a more thorough investigation of this plasmid. A first observation was that hypoxic conditions increased the relatively modest TP114 *in vitro* transfer rates to very high frequencies of conjugation *in situ*. Transposon mutagenesis coupled to conjugation experiments also highlighted the crucial role of a group of genes encoding an accessory type IVb pilus (T4P) for TP114 conjugation in the intestinal tract (Neil et al., 2020). The T4P is a structure found in I-complex plasmids (IncB/O, IncI1, IncI2, IncK, and IncZ) that was previously proposed to stabilize the mating-pair in order to allow conjugation in unstable environments (Ishiwa and Komano, 2000; Praszkier and Pittard, 2005).

## Mating-Pair Stabilization Mechanisms

**FIGURE 1:**
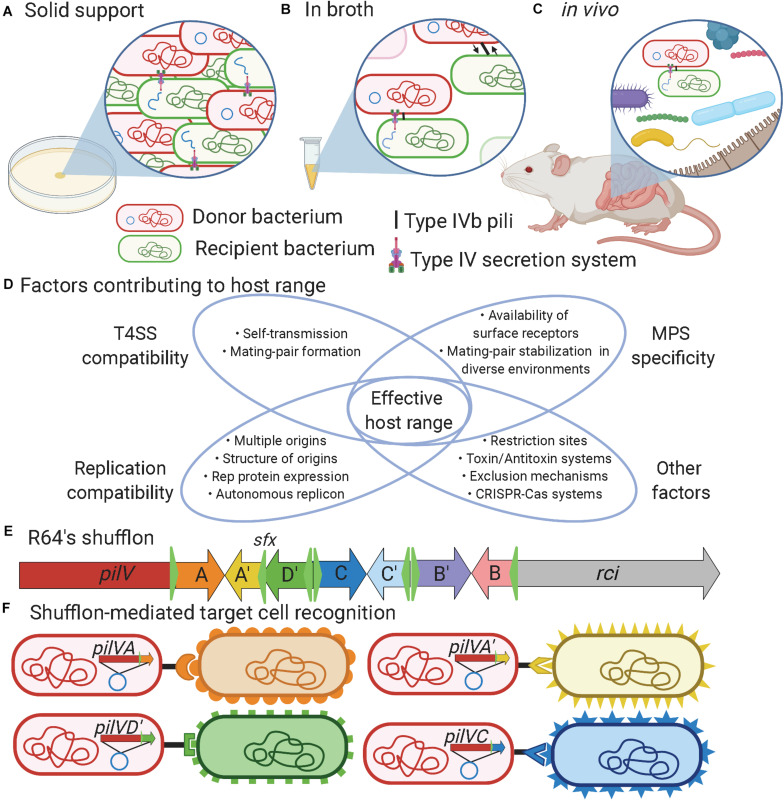
Factors influencing bacterial conjugation. **(A)** Bacterial conjugation taking place on solid support provides high cell density and close proximity between donor and recipient cells that facilitate mating-pair formation to enable plasmid transfer. **(B)** Bacteria evolving in liquid environments or **(C)** *in vivo* benefit from mating-pair stabilization (MPS) provided either by F-pili or type IVb pili to bring cells together and keep them in close contact during plasmid transfer. **(D)** Venn diagram showing the factors contributing to the effective host range of a mobile genetic element. **(E)** Schematic representation of R64’s shufflon where the C-terminus region of the *pilV* gene can undergo DNA rearrangement catalyzed by the shufflase (*rci*) to allow expression of seven variants of PilV. For example, DNA region A could be exchanged to express A′. **(F)** DNA rearrangement of the shufflon in the donor strain determines recipient specificity when mating occurred *in broth*/*in vivo* conditions. Created in BioRender.com.

The T4SS is a sophisticated nanomachine that plays an essential role in the transfer of DNA and/or protein macromolecules during bacterial conjugation. An important step during this process is mating-pair formation (MPF), which brings the donor and recipient bacteria in close contact (Chandran Darbari and Waksman, 2015; Christie, 2016; Virolle et al., 2020). In enterobacteria, two basic forms of conjugative pilus are associated with T4SS, either thin flexible or thick rigid, which influences the ability to support conjugation in liquid or solid environments (Arutyunov and Frost, 2013; Chandran Darbari and Waksman, 2015; Virolle et al., 2020). Besides MPF, a generally overlooked step called mating-pair stabilization (MPS) may be needed to keep the donor and recipient cells together long enough to allow successful DNA transfer. MPS is especially important in broth/*in vivo* conditions where bacterial mobility, flow forces, and other environmental factors could perturb the interaction between the donor and recipient cells (Clarke et al., 2008; Figures 1A–C). MPS relies on adhesins either displayed at the surface of the bacterium or on specialized pili (Hospenthal et al., 2017; González-Rivera et al., 2019). In enterobacteria, conjugative pili involved in MPS can be divided into two groups: conjugative pili and type IVb pili (T4Pb) that respectively comprise the *traN* or *pilV* adhesins (Neil et al., 2020). Additional MPS mechanisms might exist, as proposed for plasmid R6K (Neil et al., 2020), since this phenomenon remains poorly characterized in most mobile genetic elements.

Different types of conjugative pilus were reported in enterobacteria (Bradley, 1980), but the most studied is probably the F-pili (Smillie et al., 2010; Arutyunov and Frost, 2013; Koraimann, 2018). The establishment of contact between donor and recipient cells can be considered as the first rate-limiting step in conjugation as well as a key determinant for plasmid host range specificity (Virolle et al., 2020; Figure 1D). F-type pili elaborate long, thin and flexible pili that extend by polymerization of the TraA major pilin into a helical filament ranging from 1 to 20 μm in length (Christie, 2016; Koraimann, 2018). Upon contact, the F-pilus retracts, presumably by depolymerization, enabling donor cells to bring the recipient cell into close proximity for the formation of the mating pore (Clarke et al., 2008; Hospenthal et al., 2017). TraN, also named *tivF6* (Thomas et al., 2017), is an essential component for DNA transfer machinery that promotes the formation of stable donor-recipient mating-pair by interacting with OmpA or lipopolysaccharides (Klimke et al., 2005). F-pili have also been shown to promote biofilm formation, which favors plasmid transfer (Ghigo, 2001).

Type IVb pili encoded on conjugative plasmids are required only for conjugation in broth (Kim and Komano, 1997) or in the gastrointestinal tract but not on solid support (Neil et al., 2020). T4Pb are thin, flexible, helical fibers distinct from the T4SS that are mainly composed of major pilin and PilV minor adhesins that are thought to be localized to the tip of the pilus. A single motor ATPase encoding gene is predicted in T4Pb, making the extension and retraction of the pilus uncertain since two ATPases are generally present in other types of T4P (Craig et al., 2019; Ellison et al., 2019). T4Pb structures can be found encoded in all plasmid families within the I-complex (IncB/O, IncI1, IncI2, IncK, and IncZ), which were grouped based on similar morphological and serological properties of their pili (Falkow et al., 1974; Bradley, 1984; Rozwandowicz et al., 2020). The adhesin gene in I-complex plasmids is generally the last gene of the T4Pb operon and its C-terminal portion comprises a shufflon (Figure 1E). The shufflon is a dynamic DNA locus that can be re-arranged by a shufflase, encoded by *rci* (recombinase for clustered inversion), thought to be constitutively active in IncI plasmids (Brouwer et al., 2019). The shufflase recognizes specific DNA sequences called *sfx* (green triangles in Figure 1E) and promotes the recombination by inversion between two head-to-head *sfx* sites (Gyohda et al., 2002). This results in variations of the C-terminal sequence of the minor pilin PilV (Komano, 1999), thus changing the specificity of these pili to recognize different structures in lipopolysaccharides (Ishiwa and Komano, 2000) or other cell surface appendages (Figure 1F).

## Factors Influencing Conjugation in the Gut Microbiota

The gut microbiota is a complex assembly of microorganisms (Lloyd-Price et al., 2016). The high density of bacteria in this environment could thus be seen as a favorable context for conjugative elements to promote their dissemination (Norman et al., 2009). However, several factors that can act at different steps of conjugative transfer can limit the host spectrum or affect the transfer rates of conjugative elements (Figure 1D). The first barrier to bacterial conjugation in the gut is the regulation of mobile genetic element transfer genes by environmental conditions (Fernandez-Lopez et al., 2014; Getino and de la Cruz, 2018). For example, plasmid TP114 was found to be active by low oxygen concentrations (Neil et al., 2020). Many conjugative plasmids respond to specific conditions that may not be found in the gut and hence cannot reach high transfer rates in this environment (García-Quintanilla et al., 2008; Aviv et al., 2016; Neil et al., 2020). In some cases, MPS could be essential or significantly increase transfer rates by establishing and stabilizing the contact between the donor and recipient bacteria (Neil et al., 2020). For this purpose, conjugative elements may use adhesins that recognize receptors at the surface of recipient bacteria (Ishiwa and Komano, 2004). However, in certain environmental niches such as in a biofilm, the role of adhesins and MPS might not be as important, allowing the T4SS to enter in contact with potentially more diverse bacterial species (Król et al., 2013). The T4SS of the conjugative element also has to penetrate the recipient bacterium cell wall and membrane. The drastically different structures of Gram-negative and Gram-positive bacteria represent a physical barrier that is likely restraining the host range of some conjugative plasmids (Domaradskiǐ, 1985). Surface or entry exclusion represent more sophisticated mechanisms that impact conjugation (Garcillán-Barcia and De La Cruz, 2008; Arutyunov and Frost, 2013). In addition, DNA molecules that are successfully transferred must not be targeted by restriction enzymes or CRISPR-Cas systems (Wilkins, 2002; Garneau et al., 2010; Roy et al., 2020). Conjugative plasmids also have to interact with the cellular machinery of their new host to allow the expression of their genes and their maintenance. Establishing the host range of a particular conjugative element is thus a complex task that requires careful investigation of several factors (Jain and Srivastava, 2013) such as the environmental conditions, the nature of the host and recipient bacteria along with other key phenomena such as MPS, MPF, gene expression, and plasmid replication (Figure 1D).

## The Relation Between Conjugative Plasmids in the Gut

Most *in situ* conjugation studies to date have used simplified models involving a single conjugative element present in the donor bacterium (Neil et al., 2020; Ott et al., 2020). This does not necessarily represent natural conditions as gut *Enterobacteriaceae* isolates often harbor multiple plasmids (Lyimo et al., 2016; Martino et al., 2019). Mobile genetic elements were shown to have complex relationships (Getino and de la Cruz, 2018). Some conjugative plasmids, such as IncI plasmids, encode transcription factors that inhibit IncF plasmid conjugation (Gasson and Willetts, 1975, 1976, 1977; Gaffney et al., 1983; Ham and Skurray, 1989). In other cases, the regulatory proteins from a conjugative plasmid or an integrative and conjugative element (ICE) can activate gene expression in other mobile genetic elements such as genomic islands. In an elegant study, it was also shown that some mobile genetic islands such as SGI1, encodes for T4SS subunits that can reshape the mating apparatus of IncC plasmid pVCR94 to promote SGI1 self-propagation over pVCR94 conjugation (Carraro et al., 2017). SGI1 was also found to destabilize pVCR94 maintenance mechanisms. Examples of these types of relationships are plentiful, illustrating how frequent the interaction between mobile genetic elements must be in natural environments (Harmer et al., 2016).

Some plasmids, such as the P-type systems (RP4, R388, and pKM101) lack MPS and display lower conjugation rates in unstable environments such as culture broth or the gut microbiota (Chandran Darbari and Waksman, 2015; Neil et al., 2020). For instance, IncP plasmid RP4 showed no transfer in the intestinal tract in absence of antibiotic selection for the transconjugants (Licht et al., 2003). However, conjugation was shown to have implications in the stability of IncP plasmid pKJK5 in the intestinal microbiota of germ-free rats (Bahl et al., 2007). Other evidence suggests that these plasmids could highjack MPS mechanisms from other conjugative elements found in the same donor cells in a parasitic manner (Gama et al., 2017). This strategy could be beneficial to some plasmids, allowing their own transfer in a stable environment while taking advantage of other plasmids MPS systems in unstable environments. Therefore, plasmids that do not encode MPS systems should not be deemed strictly incapable of transferring in the gut microbiota. Additional work will be needed to evaluate, characterize and quantify this phenomenon and could bring new insights on the mobility of genes in the gut microbiota.

## Conclusion and Applications of this Knowledge

Bacterial conjugation can reach high transfer rates in the gut microbiota. Direct evidence suggests that MPS plays an important role in this environment but the genes that are involved in this mechanism are not encoded in all plasmid families (Neil et al., 2020). MPS has been overlooked by many groups since it is not required in classical bacterial conjugation assays on agar plates where cells are already in close contact. Plasmids encoding MPS genes could hence be seen as the most versatile conjugation machinery since they can promote DNA transfer under a wider diversity of conditions. Conjugative elements that do not encode MPS mechanisms could exploit plasmids that possess this feature to promote their dissemination. Understanding the interactions between plasmids in the gut microbiota could thus provide important insights on the dissemination of antibiotic resistance.

Alternatives to conventional antibiotics include, among other, vaccines (Scully et al., 2015), phage therapy (Ando et al., 2015; Nobrega et al., 2015), predatory bacteria (Dwidar et al., 2012), and anti-plasmid or anti-conjugation strategies (Thomas and Nielsen, 2005; Williams and Hergenrother, 2008; Oyedemi et al., 2016; Cabezón et al., 2017; Getino and de la Cruz, 2018). Inhibiting horizontal gene transfer in the intestinal microbiota will require the identification of potential drug targets. Given that MPS appears to be important for bacterial conjugation in the gut (Neil et al., 2020), strategies to limit or abolish this function could lower the spread of antibiotic resistance (Craig et al., 2019). This type of technology could be used in conjunction with antibiotic treatments or before medical procedures to limit the risk of resistance to treatment (Buelow et al., 2017).

Increased knowledge of bacterial conjugation *in situ* will also be instrumental to the development of microbiome editing technologies. Using a highly effective conjugative system, genes providing benefits to their host could be transferred and integrated into the chromosome of natural residents of the microbiota, avoiding probiotic colonization resistance (Ronda et al., 2019). This DNA mobilization technology could also be used as a CRISPR-Cas delivery vehicle (Bikard et al., 2014; Citorik et al., 2014; Yosef et al., 2015; Getino and de la Cruz, 2018; Neil et al., 2019). CRISPR could be programmed to eliminate specific bacteria causing dysbiosis, antibiotic-resistant bacteria, or pathogens, providing a precision tool for microbiome editing (Bikard and Barrangou, 2017). One could also imagine that MPS could be tuned to facilitate transfer to targeted bacterial populations while leaving other microorganisms untouched by the procedure. The study of bacterial conjugation could thus provide important knowledge that could be applicable in several aspects of the fight against antibiotic resistance.

## Author Contributions

KN, NA, and SR contributed to the initial conceptualization of the review. KN and NA did initial literature reviews and manuscript drafting. SR contributed to the literature review and extensive manuscript editing. All authors contributed to the final proofs and approved the submitted version.

## Conflict of Interest

The authors have filed a patent application for the use of conjugative plasmids for microbiome editing. KN and SR are co-founders of TATUM bioscience.
